# Overview of Subcutaneous Metastatic Melanoma

**DOI:** 10.3390/cancers13092063

**Published:** 2021-04-25

**Authors:** Nicola Mozzillo

**Affiliations:** Department of Melanoma and Soft Tissue, Istituto Nazionale dei Tumori, 80131 Naples, Italy; nikmozzillo@gmail.com

Melanoma is among the most common primary solid tumors with cutaneous and subcutaneous metastasis. Subcutaneous tissue is a common site of distant metastasis in melanoma patients and is often the first sign of advanced disease as hematogenous spread, with a serious impact on 5-year survival [1].

Diagnosis should always be suspected in melanoma patient in front of nodules usually less than 2 cm in diameter, that can occur anywhere in the body, firm, round, isolated or multiple, but pathology needs to be confirmed by fine needle aspiration or by excisional biopsy to obtain reliable confirmation about the nature and eventual mutation wild type of the lesion [2].

Usually, metastases are easily detected on physical examination, that should be implemented by US, CT, MRI, or F-18Fdg PET imaging for staging, concurring to an accurate patient selection for proper treatment.

The number of lesions, disease free interval and size of tumor have been reported to be prognostic indicators [3].

Until recently, surgical resection, when radical, was the only therapeutic strategy with some realistic chance of prolonging life and improving its quality [4,5].

Surgery nowadays remains an integral part of treatment, supported by imaging improvement, early detection, low morbidity, good quality of life, possible serial resections, no resistance with other treatments, and the correlation of general immune suppression with tumor burden [6,7].

Present day new management strategies are evolving and should be taken into consideration in front of different situations, whenever surgery alone is not indicated.

Often, subcutaneous metastases are sequential, multiple, recurring or ruptured and bleeding; in which case, systemic therapy, local intralesional therapy, radiation therapy, radiofrequency, limb perfusion or infusion can be considered as possible alternatives.

Electrochemotherapy with bleomycin or cisplatin is an effective modality of local control and palliative treatment, especially for bleeding lesions, mostly for patients too frail for other treatments [8,9].

A better understanding of the biology of melanoma, the discovery of genetic BRAF and MEK mutations in almost 50% of cases, the knowledge of immune checkpoint proteins CTLA-4 and PD-1 regulating the activity of immune cells, the relationship with the tumor microenvironment in the host, and gut microbioma as a potential contributing factor deepened the understanding of mechanisms through which tumors achieve the ability to evade immune destruction.

Inhibitors of BRAF and MEK inhibitors, monoclonal antibodies targeting CTLA-4 and PD-1 and the ability to counteract the immune system by combination immune checkpoint therapy with no restriction to respect BRAF mutation status, led to the development of new therapies that revolutionized the treatment of melanoma, leading to dramatically improved patient outcomes and introducing the possibility of long-term survival [10,11].

In cases with borderline or non-resectable subcutaneous metastases, treatment actually includes immunotherapy with checkpoint blockade and targeted therapy with BRAF and MEK inhibitors, even in a neoadjuvant modality for the timing of surgery [12].

Adjuvant targeted or immune systemic therapies should also be considered to reduce the risk of regional or systemic relapse in patients rendered disease free by any of the locoregional treatment modalities [7,13,14].

A proportion of patients will either fail to respond or relapse after a period of response to immunotherapy and resistance to either BRAF or MEK inhibition occurs in a large part of patients in about one year.

Surgery can be useful in some cases where new treatments failed to reach response or where there are mixed responses or oligoprogression.

The discussion of complicated cases by a multidisciplinary team approach can be of great value in the decisions making process and is strongly suggested.

The introduction of targeted antitumor therapy and the development of novel combinations and novel checkpoint inhibitors will provide further treatment options, broadening the therapeutic landscape for patients with locally advanced and metastatic melanoma, making surgery less morbid and more effective.

The enhanced selection of patients and the results of ongoing and future clinical trials will undoubtedly design the standard of care for patients with locally advanced melanoma at high risk.
